# Crafting an academic portfolio as a clinician-scholar: Reflections from a Next5 workshop

**DOI:** 10.4102/safp.v68i1.6316

**Published:** 2026-03-04

**Authors:** Arun Nair, Klaus B. von Pressentin, Chantelle van der Bijl

**Affiliations:** 1 Department of Family Medicine, School of Clinical Medicine, Faculty of Health Sciences, University of the Free State, Kimberley, South Africa; 2 Robert Mangaliso Sobukwe Hospital, Kimberley, Northern Cape Department of Health, Kimberley, South Africa; 3 Division of Family Medicine, Department of Family, Community and Emergency Care, Faculty of Health Sciences, University of Cape Town, Cape Town, South Africa

## Introduction

Newly qualified family physicians increasingly find themselves operating at the intersection of service delivery, education, research and clinical governance leadership. The traditional dichotomy between being a ‘full-time clinician’ and being just an ‘academic’ is no longer relevant. Current thinking and emerging consensus on faculty development motivate the growing pool of clinician-scholars who deliver high-quality patient care while generating, translating and disseminating knowledge that strengthens local primary healthcare systems.^1^ Against this backdrop, the Next5 hosted a workshop (25 participants) at the South African Academy of Family Physicians 2025 Conference focused on the practicalities of crafting an academic portfolio while maintaining a clinically grounded professional identity. This article shares reflections from the workshop and offers colleagues in their early careers a pragmatic, motivating roadmap for academic portfolio development. The authors acknowledge the workshop participants for their active engagement and contributions.

## Why does an academic portfolio matter?

An academic portfolio is more than an expanded curriculum vitae. It is a structured, longitudinal record of scholarly activity, impact and professional growth.^2^ Unlike a clinical curriculum vitae, which focuses primarily on posts held and competencies acquired, an academic portfolio foregrounds teaching philosophy and contributions, research outputs, leadership philosophy and roles within scholarly service.^3^ For family physicians, this portfolio becomes a strategic asset: it supports applications for lecturer or senior lecturer posts, research funding, promotions and collaborative opportunities, while also enabling reflective professional development.^4^

Workshop discussions emphasised that attending to portfolio developments should not be deferred until one is ‘ready for academia.’ Keeping track of teaching sessions, supervision activities, audits, quality improvement projects, presentations and publications creates momentum and reduces the cognitive load of retrospective reconstruction. Importantly, portfolio development helps clinicians recognise that everyday practice activities – when framed, evaluated and disseminated – are legitimate scholarly contributions.

## The anatomy of a balanced academic portfolio

Workshop discussions highlighted four interlinked portfolio domains: research, teaching, leadership and service. Research portfolios may begin modestly through audits, case studies and quality improvement projects that address locally relevant clinical questions.^5^ Teaching portfolios should articulate a personal teaching philosophy and document teaching activities, supervision roles, curriculum contributions and learner feedback.^6^ Leadership and service portfolios capture contributions to clinical governance, professional bodies, and community engagement, making the academic value embedded in daily clinical practice visible.

## From dreaming to doing: Academic progression and the PhD pathway

Doctoral studies represent one of several options to advance your academic career, and taking on a PhD should be viewed as a significant commitment and investment. Having a PhD increasingly provides a competitive edge in securing academic positions, funding and leadership roles. If one applies the hero’s journey framework to Doctoral education, the PhD offers an opportunity to prepare for leadership and knowledge generation in family medicine.^7^ However, academic growth is incremental and cumulative, not contingent on a single credential, and depends on clarity of purpose, early mentorship, realistic timelines and institutional support.^1,6^ Viewed through this lens, further research development will add value, as doctoral training does not apply to everyone. This inclusive approach to building a pipeline for clinician-scholar researchers strengthens both individual academic identity and the discipline’s collective knowledge base.

Effective portfolios depend on scholarly visibility. Strategic journal selection, strict adherence to author guidelines and constructive engagement with peer review enhance research quality and impact.^5^ Establishing a research identity using persistent identifiers, such as ORCID, improves attribution and discoverability. Collaboration through professional networks, multidisciplinary teams, and regional research consortia such as the PRIMAFAMED and WONCA Africa networks amplifies scholarly reach while distributing workload and expertise.^8^

## Ten practical recommendations for portfolio development

Hartl and Marquez outlined 10 key recommendations for clinician-scholars who are in their early careers: start documenting early, align activities with long-term career goals, combine clinical work with scholarship pursuits, actively seek mentors and sponsors, focus on quality rather than quantity, collaborate intentionally, regularly reflect on progress, make academic impact visible, review and update the portfolio annually, and stay authentic by aligning academic efforts with personal values and community involvement needs.^9^

## Conclusion

Developing an academic portfolio is not a parallel career track but an integrated way of practising family medicine. The identity of a clinician-scholar emerges through deliberate documentation, mentorship, collaboration and reflection over time. By recognising the scholarly potential embedded in everyday clinical practice and starting early, family physicians can cultivate sustainable academic identities that enhance career progression, strengthen primary healthcare systems and contribute meaningfully to the discipline’s intellectual leadership. The Next5 workshop experience affirms that academic growth is not just reserved for a select few but also an achievable, cumulative journey open to all who choose to engage intentionally with their work.
